# A species-specific nucleosomal signature defines a periodic distribution of amino acids in proteins

**DOI:** 10.1098/rsob.140218

**Published:** 2015-04-08

**Authors:** Luis Quintales, Ignacio Soriano, Enrique Vázquez, Mónica Segurado, Francisco Antequera

**Affiliations:** Instituto de Biología Funcional y Genómica, Consejo Superior de Investigaciones Científicas (CSIC)/Universidad de Salamanca, Campus Miguel de Unamuno, 37007 Salamanca, Spain

**Keywords:** nucleosomes, protein composition, genome evolution

## Abstract

Nucleosomes are the basic structural units of chromatin. Most of the yeast genome is organized in a pattern of positioned nucleosomes that is stably maintained under a wide range of physiological conditions. In this work, we have searched for sequence determinants associated with positioned nucleosomes in four species of fission and budding yeasts. We show that mononucleosomal DNA follows a highly structured base composition pattern, which differs among species despite the high degree of histone conservation. These nucleosomal signatures are present in transcribed and non-transcribed regions across the genome. In the case of open reading frames, they correctly predict the relative distribution of codons on mononucleosomal DNA, and they also determine a periodicity in the average distribution of amino acids along the proteins. These results establish a direct and species-specific connection between the position of each codon around the histone octamer and protein composition.

## 2. Introduction

Nucleosomes facilitate the packaging of the genome inside the nucleus and modulate the access of regulators to the DNA molecule. In addition, histones can harbour a large variety of posttranslational modifications that play an essential role in genome regulation [1]. Nucleosome positioning along the genome depends on the combined contribution of several factors. For example, ATP-dependent nucleosome remodellers improve nucleosome positioning around the transcription start sites in chromatin reconstitution experiments performed *in vitro* [2] and are essential for maintaining the organization of the nucleosomal pattern *in vivo* [3–6]. As regards the contribution of transcription factors to nucleosome positioning, comparative analysis of the closely related *Saccharomyces cerevisiae* and *Saccharomyces paradoxus* species has shown that shifts in nucleosomal arrays between orthologous genes are associated with differences in the size of the nucleosome depleted region (NDR) at their promoters, suggesting that factors bound to them could act as the border elements postulated in the statistical positioning model [7,8]. A third factor contributing to nucleosome positioning is the DNA sequence itself. The strong bending imposed on the double helix due to its tight association with the histone octamer [9,10] means that the affinity between histones and DNA varies depending on the different flexibility of dinucleotides in mononucleosomal DNA [11]. AA and TT dinucleotides favour bendability and have been reported to be distributed on mononucleosomal DNA with the 10-bp periodicity of the helical repeat of DNA [12–15]. More recent studies have described a similar periodicity in other dinucleotides that either contributes or disfavours nucleosome positioning, such that different combinations could modulate the interaction between specific nucleosomes and DNA [16].

In *S. cerevisiae* and *Schizosaccharomyces pombe*, the biological outcome of all the factors contributing to nucleosome positioning is that approximately 80% of their genomes are organized in positioned nucleosomes. Such a pattern remains largely invariable under a broad range of transcription rates and also during meiosis, despite the major structural processes undergone by the chromosomes [17,18].

Based on the extensive positioning of nucleosomes in yeast genomes [18–22], we have searched for sequence determinants associated with positioned nucleosomes in four species of fission and budding yeasts. We found that the distribution of the four mononucleotides along mononucleosomal DNA follows a species-specific pattern, which in the case of open reading frames (ORFs) overlaps with the distribution of amino acids in proteins.

## 3. Material and methods

### 3.1. Strains and growth conditions

Genomic nucleosome maps of asynchronous exponential *S. pombe* wild-type 972h^−^ cells have been reported previously [18]. Nucleosome maps of *Schizosaccharomyces octosporus* CBS1804 and *Schizosaccharomyces japonicus* var. *japonicus ade12*^−^ FY53 were generated from 400 ml cultures grown in rich medium (YES) at 32°C up to a density of 1.5 × 10^7^ cells ml^−1^. Nucleosome maps of *S. cerevisiae* W303-1a were generated from cultures grown in 200 ml of rich medium (YEPD) at 30°C up to a density of 10^7^ cells ml^−1^.

### 3.2. Preparation of mononucleosomal DNA

Mononucleosomal DNA was isolated as described [23]. The amount of Zymolyase 20 T used to prepare spheroplasts was optimized experimentally for each species to generate an 80 : 20 ratio of mononucleosomes to dinucleosomes, as described in [24]. Cell suspensions of cultures of *S. octosporus* and *S. japonicus* were treated with 5 mg ml^−1^ and 1.2 mg ml^−1^ of Zymolyase 20 T, respectively, for 30 min at 30°C. Spheroplasts were treated with 200 units ml^−1^ of micrococcal nuclease at 37°C for 45 min. Cells of *S. cerevisiae* were treated with 0.5 mg ml^−1^ of Zymolyase 20 T for 10 min at 30°C. Permeabilized cells were treated with 45 units ml^−1^ of micrococcal nuclease at 37°C for 10 min. Mononucleosomal DNA was recovered from 1.5% agarose gels.

### 3.3. Sequencing and alignment of sequence reads

Mononucleosomal DNA was sequenced with an Illumina Genome Analyzer IIx using the single-read sequencing protocol. A total of 18 261 406 (36 bp) (taken from [18]), 17 901 356 (40 bp), 11 817 458 (40 bp) and 18 269 690 (40 bp) sequence reads were aligned to the *S. pombe*, *S. octosporus*, *S. japonicus* and *S. cerevisiae* genomes, respectively, using BOWTIE 1.0.0 [25] with two mismatches permitted, and multireads were discarded. This represents a genome average coverage of 52-, 62-, 42- and 59-fold. The following reference genomes were used for the alignments: *S. pombe* (ASM294v2.20, assembly 13 August 2013) from PomBase; *S. octosporus* (SO6, assembly 7 June 2012) and *S. japonicus* (SJ5, assembly 7 June 2012) from the Broad Institute *Schizosaccharomyces* group Database, and *S. cerevisae* strain S288C (R64-1-1, assembly 3 February 2011) from the *Saccharomyces* Genome Database.

### 3.4. Generation of genomic nucleosome occupancy maps

After mapping the sequence reads to the corresponding reference genomes, the signals for each strand were smoothed using a five-level one-dimensional discrete biorthogonal 3.1 wavelet (bior3.1) decomposition and an additional multilevel reconstruction of the signal using only the approximation coefficients [26]. The de-noised profile facilitates the straightforward identification of individual peak maxima using a simple hill-climbing method. To estimate the value for the shifting of signal between both strands, we calculated the average distance between peaks from the complementary strands that corresponded to the boundaries of the same individual nucleosomes. Only peaks from each strand along the genome whose height was higher than twice the genome-wide mean depth coverage and that mapped at least 100 nucleotides away from other peaks of the same height were selected. Next, the original signal profile of the complementary strands was shifted in the 3′ direction for both strands by half of the previous calculated distance to generate a first version of the nucleosome occupancy map. The resulting signal was smoothed using the same wavelet process described above and was normalized relative to the average genome-wide depth coverage to generate the final nucleosome occupancy map. This protocol has been recently incorporated into a bioinformatic tool based in wavelets (NUCwave) for the automatic generation of nucleosome occupancy maps [27].

### 3.5. Identification of well-positioned nucleosomes

After wavelet-smoothing, the centre of well-positioned nucleosomes was defined as peak positions whose level of occupancy was above the genome average occupancy and the nearest maximum on each direction was at least 120 nucleotides away. According to this criterion, we selected the following mononucleosomal DNA sequences from nucleosomes in the whole genome, in ORFs and in intergenic regions (IGRs), respectively, in the four species: *S. pombe*: 38 154, 18 629 and 4581; *S. octosporus*: 46 120, 23 657 and 5091; *S. japonicus*: 27 074, 13 085 and 2963; *S. cerevisae*: 34 526, 21 918 and 5277.

### 3.6. Generation of mono-, di-, trinucleotide and amino acid profiles

**Figure 1.**
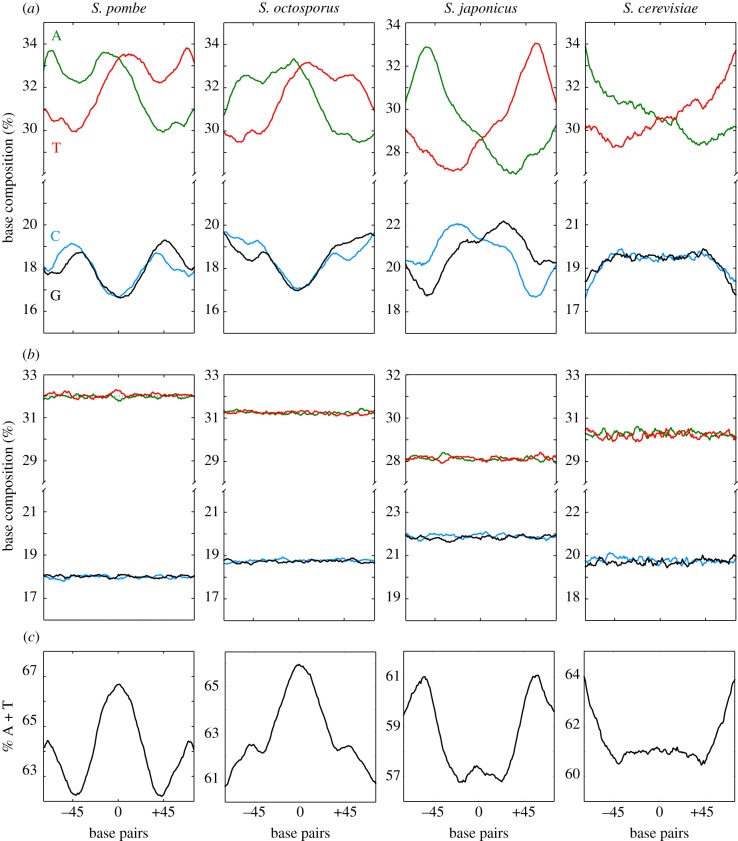
Patterns of nucleotide distribution across mononucleosomal DNA. (*a*) Base composition profiles of the four nucleotides across 38 154, 46 120, 27 024 and 34 526 mononucleosomal DNA sequences from the whole genome of *S. pombe, S. octosporus, S. japonicus* and *S. cerevisiae*, respectively, aligned to their central position. (*b*) Profiles of the same number of DNA fragments of the same length as in (*a*) selected at random from the genome of each species. (*c*) A + T content of the mononucleosomal DNA sequences shown in (*a*). The *x*-axis indicates positions relative to the centre of mononucleosomal DNA.

**Figure 2.**
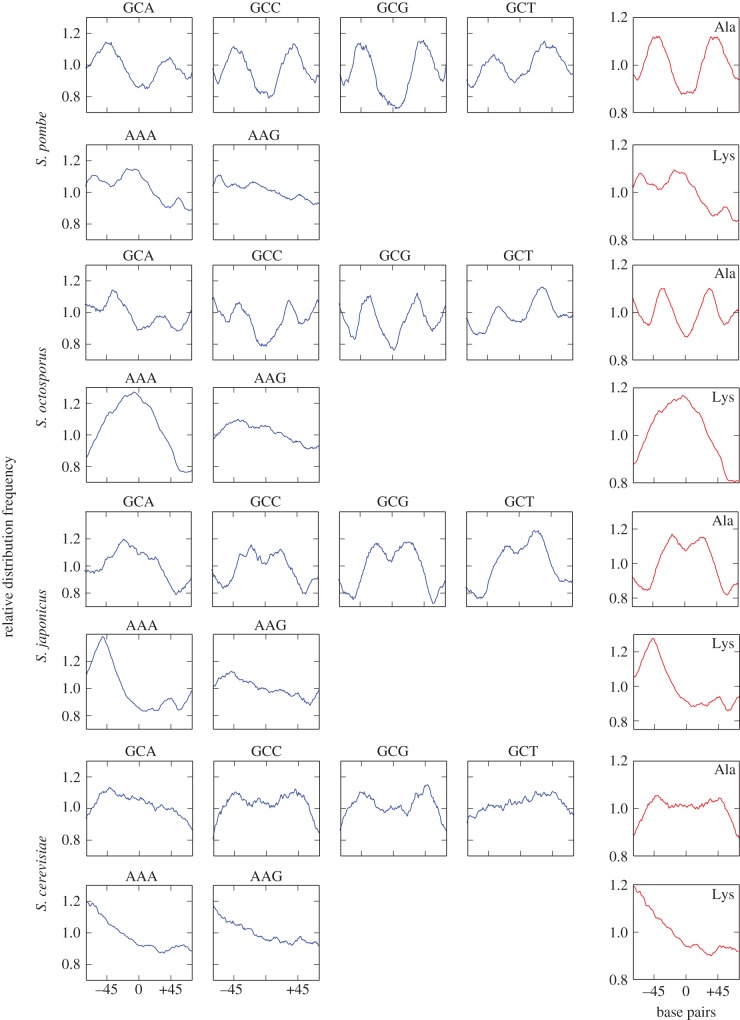
Trinucleotide profiles of mononucleosomal DNA. The frequencies of trinucleotides across mononucleosomal DNA (blue) corresponding to the codons for alanine and lysine in *S. pombe, S. octosporus, S. japonicus* and *S. cerevisiae* were grouped in aggregated profiles (red). The *y*-axis indicates the relative frequency of each trinucleotide (blue) or their aggregated value (red) normalized to the genomic average. The *x*-axis represents the distance relative to the central position of mononucleosomal DNA. The complete set of profiles for the 61 coding codons corresponding to the 20 amino acids in the four species is shown in the electronic supplementary material, figure S6.

**Figure 3.**
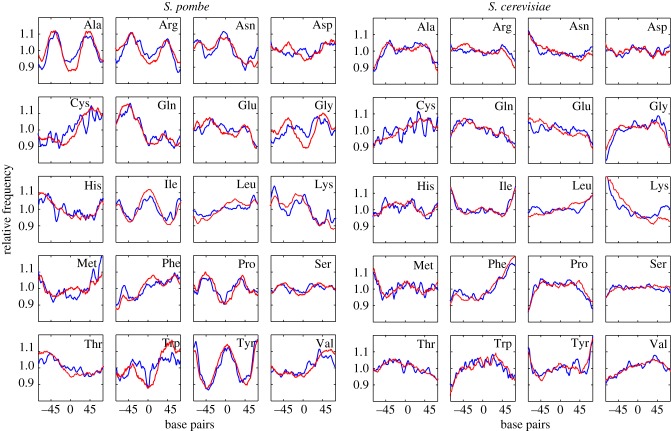
Trinucleotide profiles predict the distribution of amino acids encoded by mononucleosomal DNA. The aggregated profile of trinucleotides corresponding to codons for the 20 amino acids (red) coincides with the actual profile of amino acid distribution in proteins (blue) in *S. pombe* and *S. cerevisiae*. The *y*-axis indicates the relative frequency of aggregated trinucleotides (red) normalized to the average genomic composition and the relative frequency of amino acids (blue) encoded by mononucleosomal DNA in ORFs. The *x*-axis represents the distance relative to the central position of mononucleosomal DNA. Results for *S. octosporus* and *S. japonicus* are shown in the electronic supplementary material, figure S7.

**Figure 4.**
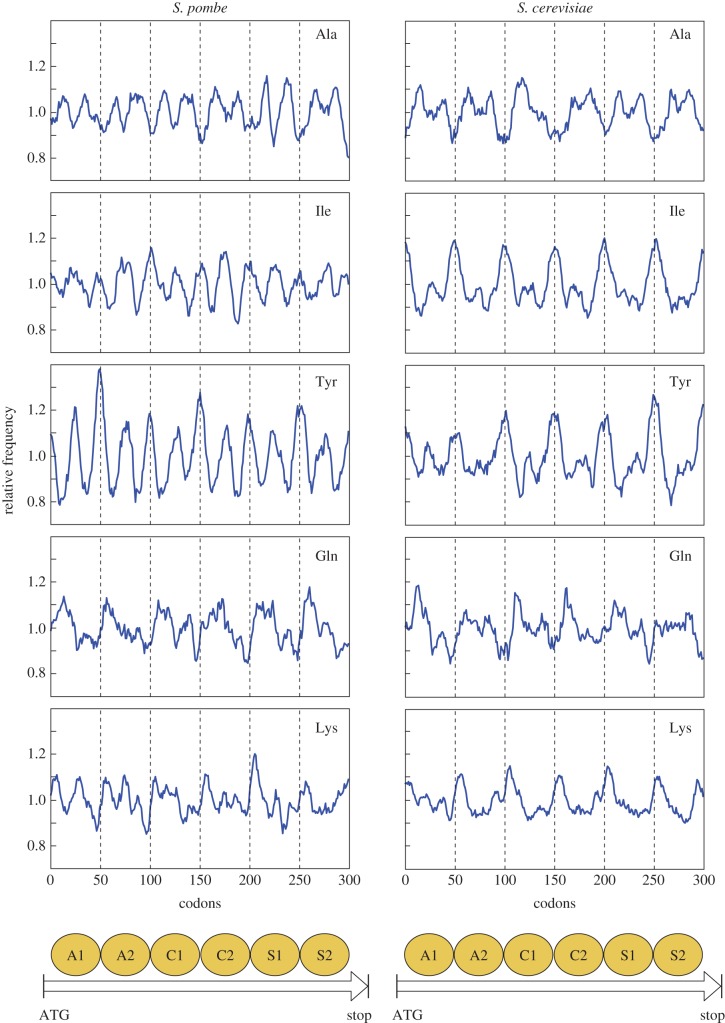
Periodic distribution of amino acids along ORFs. The same average distribution of amino acids encoded by mononucleosomal DNA from different nucleosomes generates a periodic profile along ORFs. Small differences between nucleosomes at different positions are due to the fact that the number of sequences analysed in each of the six cases is lower than in figure 3. The *y*-axis indicates the relative frequency of amino acids. The *x*-axis indicates the number of codons along the six groups of mononucleosomal DNA. The bottom diagram represents the position of the two nucleosomes immediately downstream from the ATG codon (A1 and A2) and another two mapping to the centre of ORFs (C1 and C2) or immediately upstream from the STOP codon (S1 and S2). Arrows represent their position along a generic ORF.

Mononucleosomal sequences 150 bp long associated with well-positioned nucleosomes were aligned to the nucleosome midposition (dyad). The frequencies of mononucleotides (figure 1 and electronic supplementary material, figure S2) were calculated for each position. The frequencies of di- (electronic supplementary material, figure S4) and trinucleotides (figure 2 and electronic supplementary material, figures S5 and S6) and those of the sum of trinucleotides corresponding to codons for each amino acid (figures 2 and 3, and electronic supplementary material, figures S6 and S7) were also calculated for each position and normalized to the corresponding genome averages. All frequencies were represented using a smoothing window of nine nucleotides and a step of one nucleotide. The amino acid profiles in coding regions (figure 3 and electronic supplementary material, figures S7 and S9) were represented using a smoothing window of three codons and a step of one codon. For the ORF analysis in figure 4, the same analysis was performed on 1549 (*S. pombe*) and 2046 (*S. cerevisiae*) mononucleosomal DNA sequences corresponding to each of the six groups of nucleosomes indicated in the text.

## 4. Results

### 4.1. Species-specific nucleotide patterns in mononucleosomal DNA

To analyse the nucleotide composition of mononucleosomal DNA, we initially selected 38154 DNA sequences 150-bp long from well-positioned nucleosomes in the *S. pombe* genome and aligned them to their central position to calculate the percentage of the four nucleotides at each of the 150 positions. Figure 1*a* shows that the distribution of the four mononucleotides followed a highly structured profile, with strong asymmetry in the distribution of adenine (A) and thymine (T) in the same DNA strand relative to the dyad position. The fact that the A and T profiles mirrored each other in the same DNA strand implied that they were palindromic in the two strands of DNA. The same applied to the cytosine (C) and guanine (G) profiles although they showed a lower degree of asymmetry than A and T. As a control that these patterns were strictly associated with mononucleosomal DNA, the alignment of another set of 38 154 sequences 150-bp long selected at random along the *S. pombe* genome generated a flat profile, in which the nucleotide composition coincided with the average genome content (figure 1*b*).

To determine whether these nucleosomal signatures were also present in other genomes, we generated nucleosomal maps (electronic supplementary material, figure S1) of *S. octosporus* and *S. japonicus*, which diverged from *S. pombe* 119 and 221 Ma, respectively [28], and from *S. cerevisiae*, whose phylogenetic distance from *S. pombe* is comparable to that between either of them and mammals [29]. The analysis of mononucleosomal DNA sequences from these species also showed well-defined asymmetrical and palindromic nucleotide patterns, although their individual shapes were different among them and also relative to *S. pombe* (figure 1*a*). As in *S. pombe*, the alignment of sequences 150-bp long chosen at random from their genomes generated flat profiles that coincided with the average genome composition of each species (figure 1*b*). The differences in the nucleosomal signatures among the four species are further highlighted in the aggregated representation of the A + T content along mononucleosomal DNA (figure 1*c*).

To check whether nucleosomal signatures were present in transcribed and non-transcribed regions, we independently analysed mononucleosomal DNA sequences mapping to IGRs and to ORFs in *S. pombe* and *S. cerevisiae*. Comparable profiles were detected in both cases (electronic supplementary material, figure S2), although the A and T content was lower in ORFs than in IGRs, in agreement with the different overall base composition of both types of region in the genome. In the case of ORFs, the A + T profile was maintained in the three positions of the 150 codons along mononucleosomal DNA (electronic supplementary material, figure S3), indicating that it could not be accounted for by the higher sequence degeneracy of the third codon position in the genetic code.

Well-defined and asymmetric patterns, consistent with those of the four mononucleotides in figure 1, were also observed in the distribution of dinucleotides (electronic supplementary material, figure S4) and trinucleotides (electronic supplementary material, figure S5). Their palindromic distribution in the two strands of DNA is clearly shown by the mirrored distribution of the reverse complementary di- and trinucleotides (blue and red diagrams in electronic supplementary material, figures S4 and S5).

### 4.2. Genome-wide nucleosomal signatures parallel a periodic distribution of amino acids in proteins

Since nucleosomal signatures are present in non-transcribed and coding regions (electronic supplementary material, figure S2), we wondered whether these genome-wide trinucleotide patterns would have any impact on the distribution of amino acids in proteins. To test this possibility, we generated the profiles of the 64 trinucleotides from mononucleosomal DNA (electronic supplementary material, figure S6, blue) and grouped them on the basis of their identity with the codons for each of the 20 amino acids in *S. pombe*, *S. octosporus*, *S. japonicus* and *S. cerevisiae* (electronic supplementary material, figure S6, red). It is important to note that these profiles were generated directly from the distribution of trinucleotides on genomic mononucleosomal DNA independently from the distribution of codons along ORFs. The individual and aggregated profiles of the trinucleotides corresponding to the codons of alanine and lysine in the four species are shown in figure 2.

To test whether there would be some connection between the trinucleotide profiles and the actual distribution of amino acids along ORFs, we generated the amino acid profiles of mononucleosomal DNA fragments 150-bp long derived exclusively from ORFs in the four species (see Material and methods). Figure 3 shows that the codon distribution profile predicted by the frequency of trinucleotides (red line) matched very closely to the actual distribution of the 20 amino acids encoded by mononucleosomal DNA in *S. pombe* and *S. cerevisiae* (blue line). The distribution of the 20 amino acids in *S. octosporus* and *S. japonicus* is shown in the electronic supplementary material, figure S7. The distribution of some amino acids encoded by A + T-rich codons, such as tyrosine (TAC/TAT), was symmetrical and comparable with the A + T mononucleosomal pattern (figure 1*c*), in sharp contrast with the reverse profile of amino acids encoded by G + C-rich codons, such as alanine (GCN) (figure 3). Owing to the strong asymmetry in the distribution of A and T in each DNA strand (figure 1*a*), the amino acids encoded by A-rich codons such as lysine (AAA/AAG) or glutamine (CAA/CAG) followed a skewed distribution which was the reverse of that of T-rich codons like phenylalanine (TTT/TTC) and cysteine (TGT/TGC) (figure 3).

Recent comparative studies have shown that nucleosome mapping by MNase digestion using the single-read or paired-end sequencing protocols or by chemical cleavage of DNA at the dyad region generates comparable nucleosome maps in *S. cerevisiae* [27,30]. In agreement with these observations, figure S8 in the electronic supplementary material shows that the three approaches generate very similar maps as regards the position of individual nucleosomes along the genome in *S. pombe* and *S. cerevisiae*. Consistent with this scenario, figure S9 in the electronic supplementary material shows that the amino acid profiles along mononucleosomal DNA of ORFs of the two yeasts independently identified by the three methods are comparable. This degree of concordance indicates that nucleosomal signatures are a robust feature of yeast genomes that is detectable independently of the experimental approach used to map the nucleosomes.

The aggregated pattern of codon distribution in mononucleosomes (figure 3 and electronic supplementary material, figure S7) raised the question of whether the same distribution would be present in all the nucleosomes along the coding regions. To test this possibility, we extracted the mononucleosomal sequences underlying six mutually exclusive groups of nucleosomes at different positions along the ORFs and determined their codon distribution profile (figure 4). The groups included the first and second nucleosomes immediately downstream from the ATG codon (A1 and A2), the two nucleosomes closer to the central coordinate of the ORF (C1 and C2) and the two nucleosomes immediately upstream from the STOP codon (S1 and S2) of 1549 and 2046 ORFs in *S. pombe* and *S. cerevisiae*, respectively. Figure 4 shows that, indeed, the species-specific average pattern of amino acid distribution was present in all the nucleosomes along the ORF, which resulted in an oscillating and periodic profile along its length. Taken together, these results show that nucleosomal signatures across the genome are paralleled by a periodic average distribution of amino acids in proteins, depending on where their corresponding codons are located relative to the dyad around the nucleosome core.

## 5. Discussion

Several studies have described a link between the nucleosomal organization of the genome and a periodic variation in base composition or in the frequency of polymorphisms in DNA [31–36]. The debate is still open as to whether these oscillating sequence profiles have been selected by their contribution to nucleosome positioning or whether they are a consequence of the differential stability of the DNA molecule around the histone core [37–39]. A role for selection is supported by the detailed comparison between the intra- and intergenic rates of sequence divergence around nucleosomal dyads in primates [39]. This analysis detected signs of positive and negative selection in the maintenance of a higher and a lower than average G + C content in the dyad and linker regions, respectively. Similarly, the finding that the linker DNA across genes in *S. cerevisiae* evolves approximately 6% slower than core DNA sequences led to the proposal that codons rich in A and T could have been selected in linker sequences owing to their contribution to excluding nucleosomes [40].

Other studies have pointed out that the different rates of divergence and base composition between linker and core mononucleosomal DNA could be due to a differential stability of the DNA sequence around the histone core [33,37–39]. This possibility is consistent with the fact that the mutational spectrum is not uniform along mononucleosomal DNA in *S. cerevisiae*, where the substitution rate is higher than the genome average in the dyad region and gradually declines to a rate lower than average at both ends of mononucleosomal DNA [33]. Interestingly, the central region shows the strongest DNA–histone interaction, as measured by mechanical unzipping of DNA molecules complexed with single nucleosomes *in vitro* [41]. However, the selective or mutational origins of the nucleosomal signatures are not mutually exclusive. It is conceivable that structural differences in histone octamers, repair complexes or other chromatin proteins among species could determine a different rate or bias of mutation or repair between different mononucleosomal DNA regions [33]. This non-uniform mutational landscape is compatible with the selective fixation of mutations favourable to stabilizing DNA–histone interactions.

As regards the biological significance of nucleosomal signatures, it is important to note that they represent a genome-wide phenomenon (electronic supplementary material, figures S1 and S2) whose influence on the amino acid composition of proteins is evidenced by their potential to predict the relative distribution of codons along mononucleosomal DNA (figure 3 and electronic supplementary material, figure S7). The different profiles among species are likely to increase protein diversity and could explain, for example, the paradox that the high conservation of gene content, gene order and gene structure among the three species of *Schizosaccharomyces* studied here does not match the degree of divergence between the amino acid composition of their proteins [28].

The diversity of nucleosomal signatures could contribute to explaining the long known observation that the same DNA is packed differently by nucleosomes of a different species (e.g. [42–45]). Along the same lines, nucleosomal signatures could also be very relevant for the interpretation of many structural *in vitro* analyses of DNA–histone interactions where synthetic or repetitive DNA molecules, or even the entire genome of an organism, are reconstituted *in vitro* with histones from a different species [46].

## Supplementary Material

Suplementary Figures 1_9

## Data accessibility

The nucleosome sequencing data have been deposited in the GEO database under the accession number GSE52170.
