# Supplementary material for: A species-specific nucleosomal signature defines a periodic distribution of amino acids in proteins
Source: Open Biol. 2015 Apr 8;5(4):140218. doi: 10.1098/rsob.140218 (PMC4422121; doi:10.1098/rsob.140218)
Supplement: Suplementary Figures 1_9 [file rsob140218supp1.pdf]

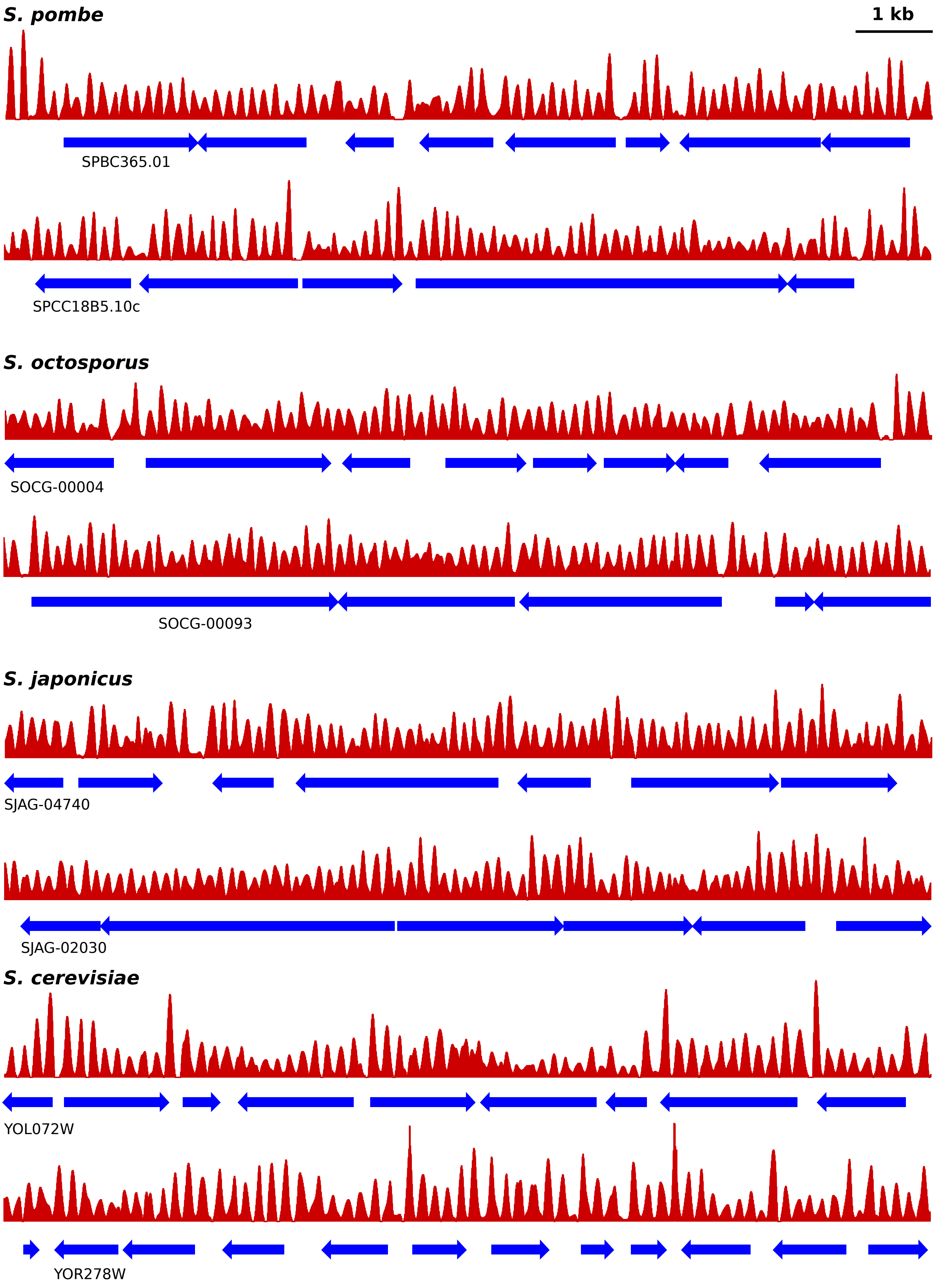

Supplementary Figure 1

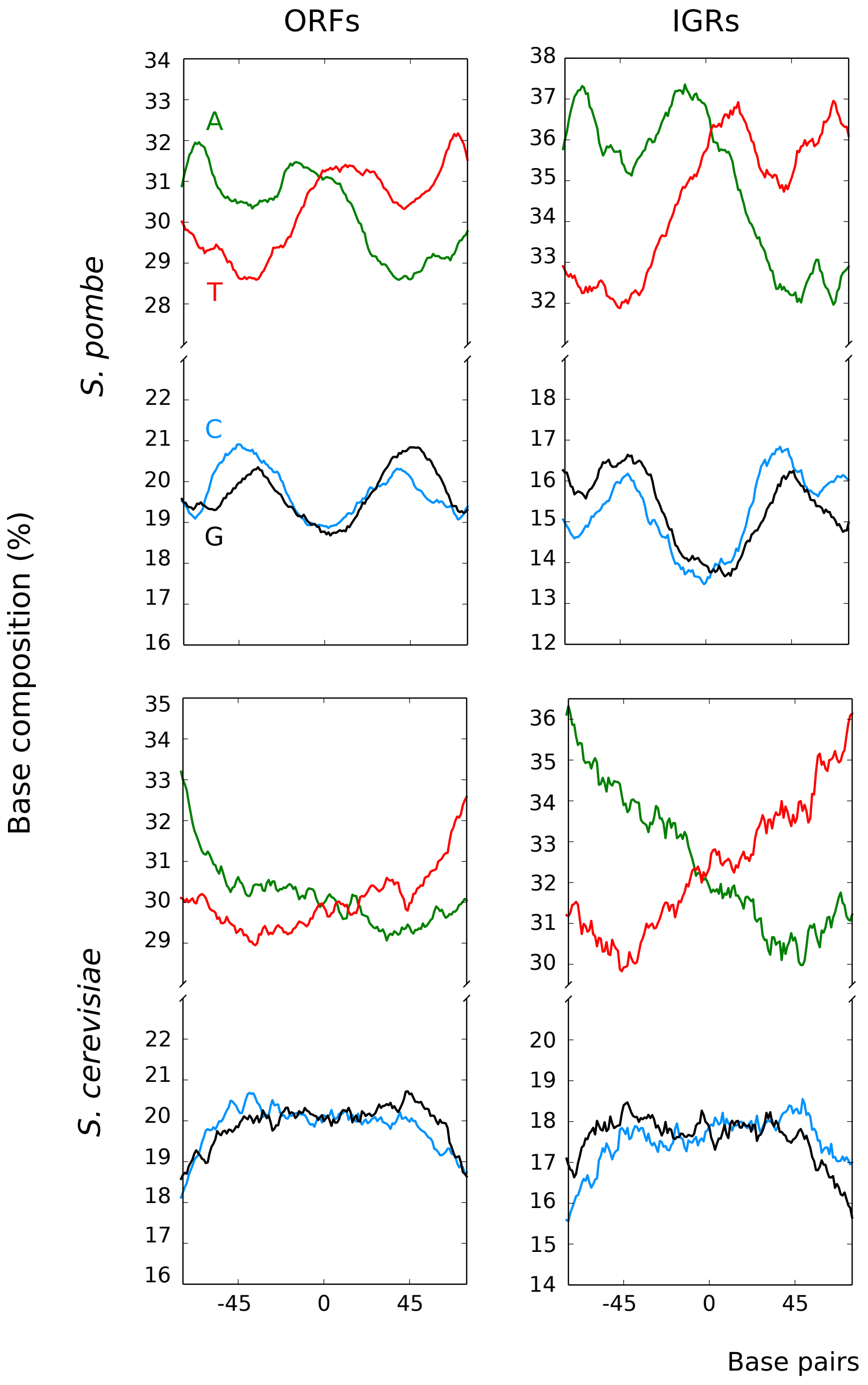

Supplementary Figure 2

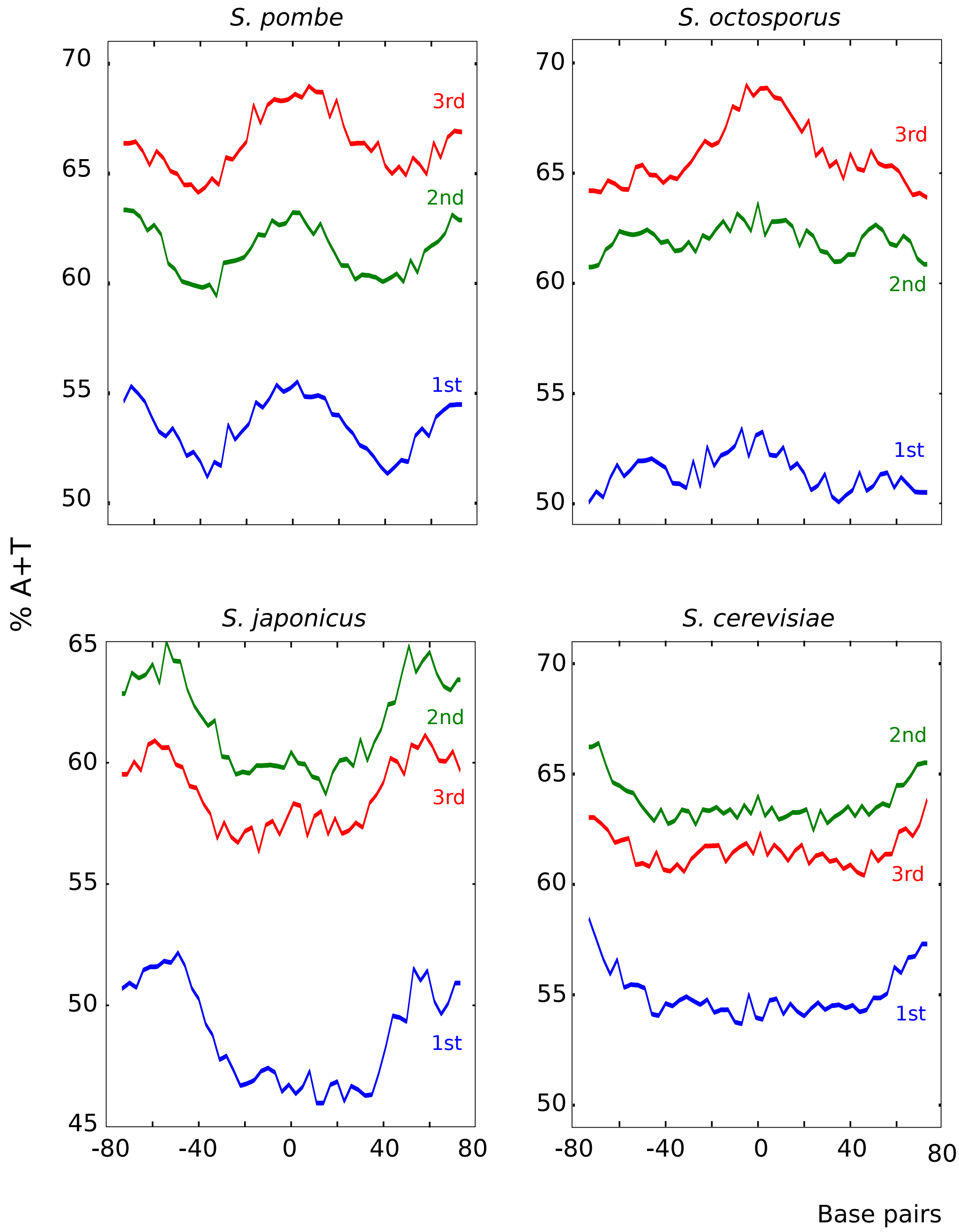

Supplementary Figure 3

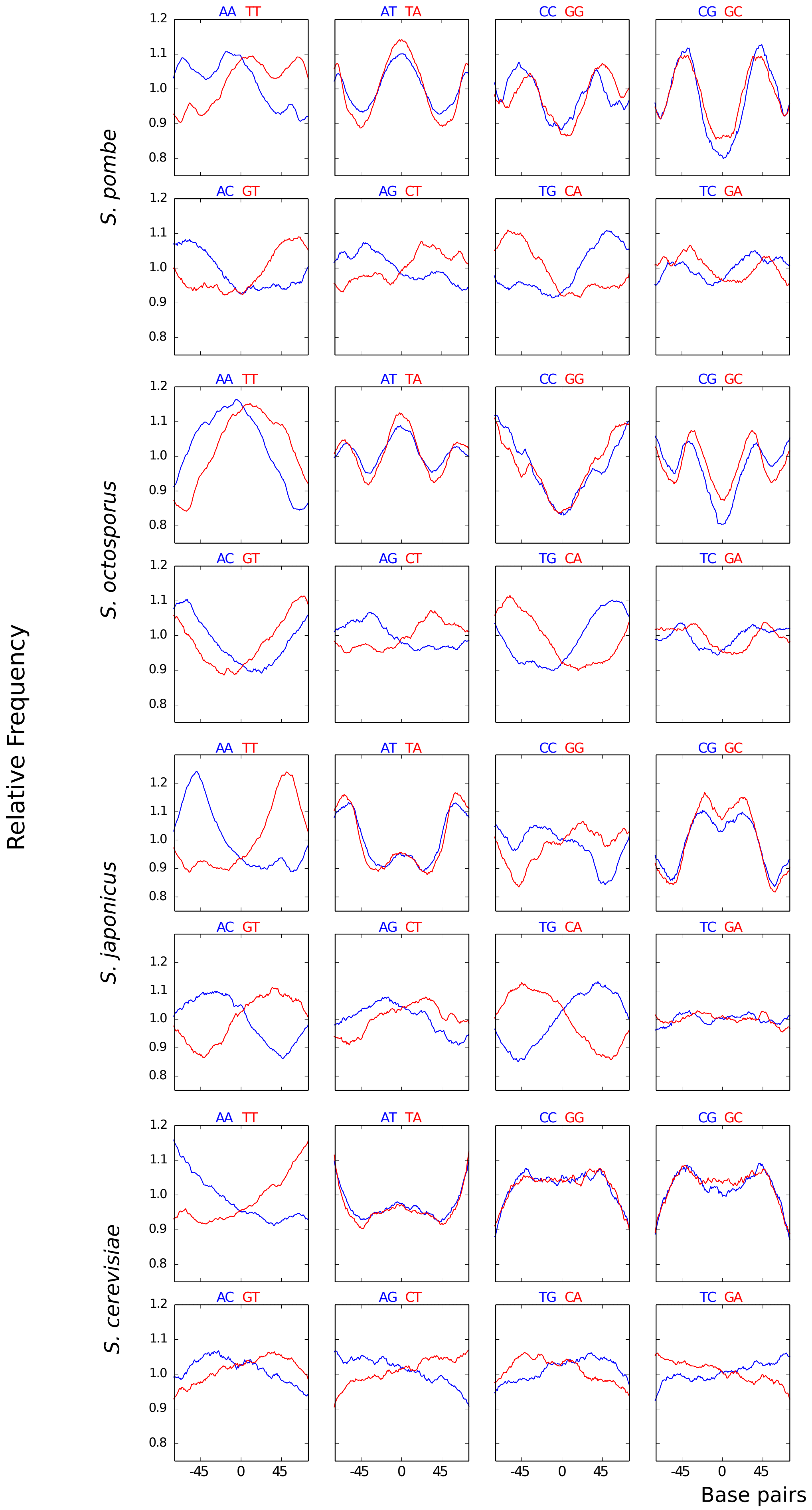

Supplementary Figure 4

S. pombe

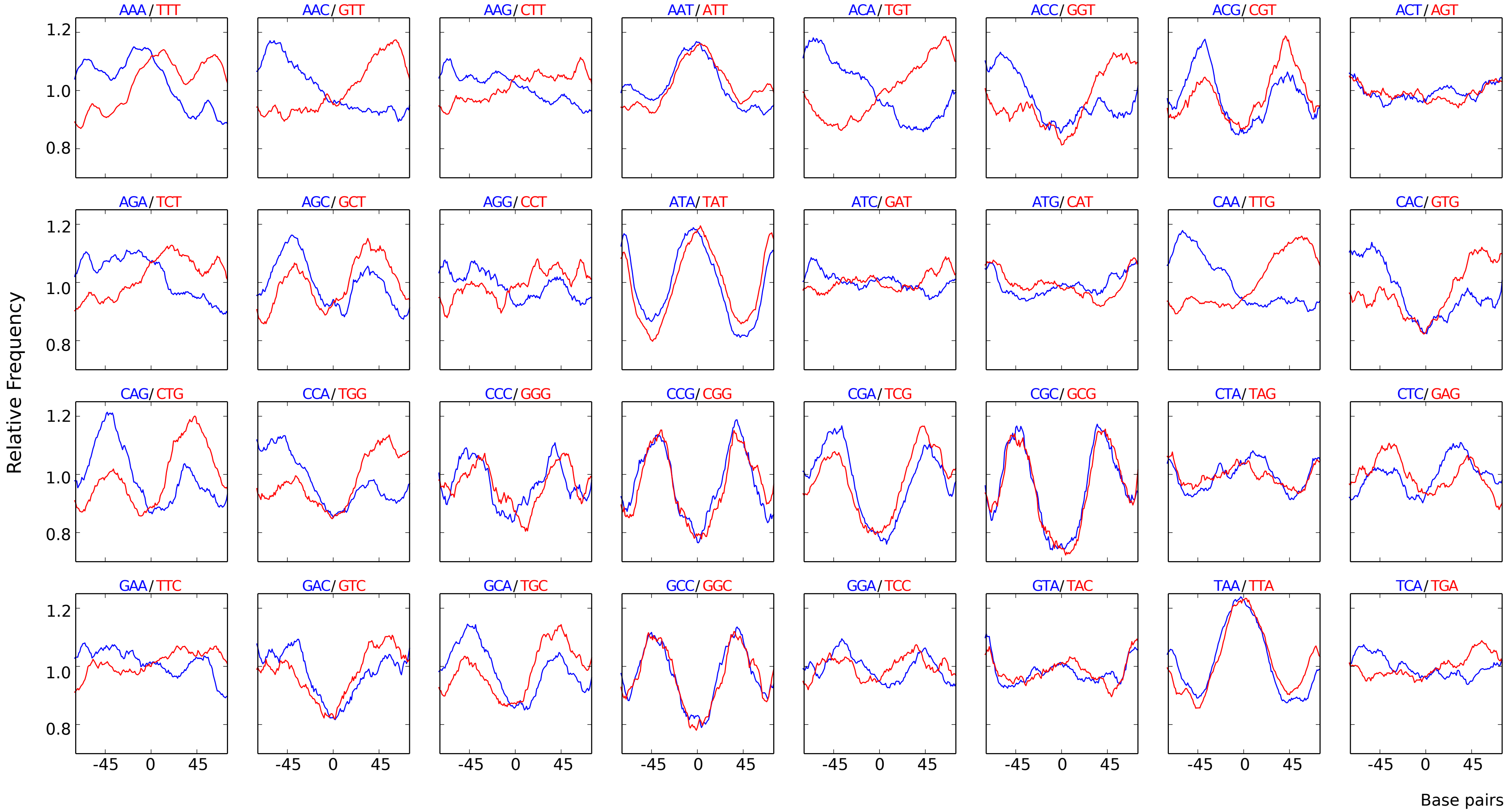

Supplementary Figure 5A

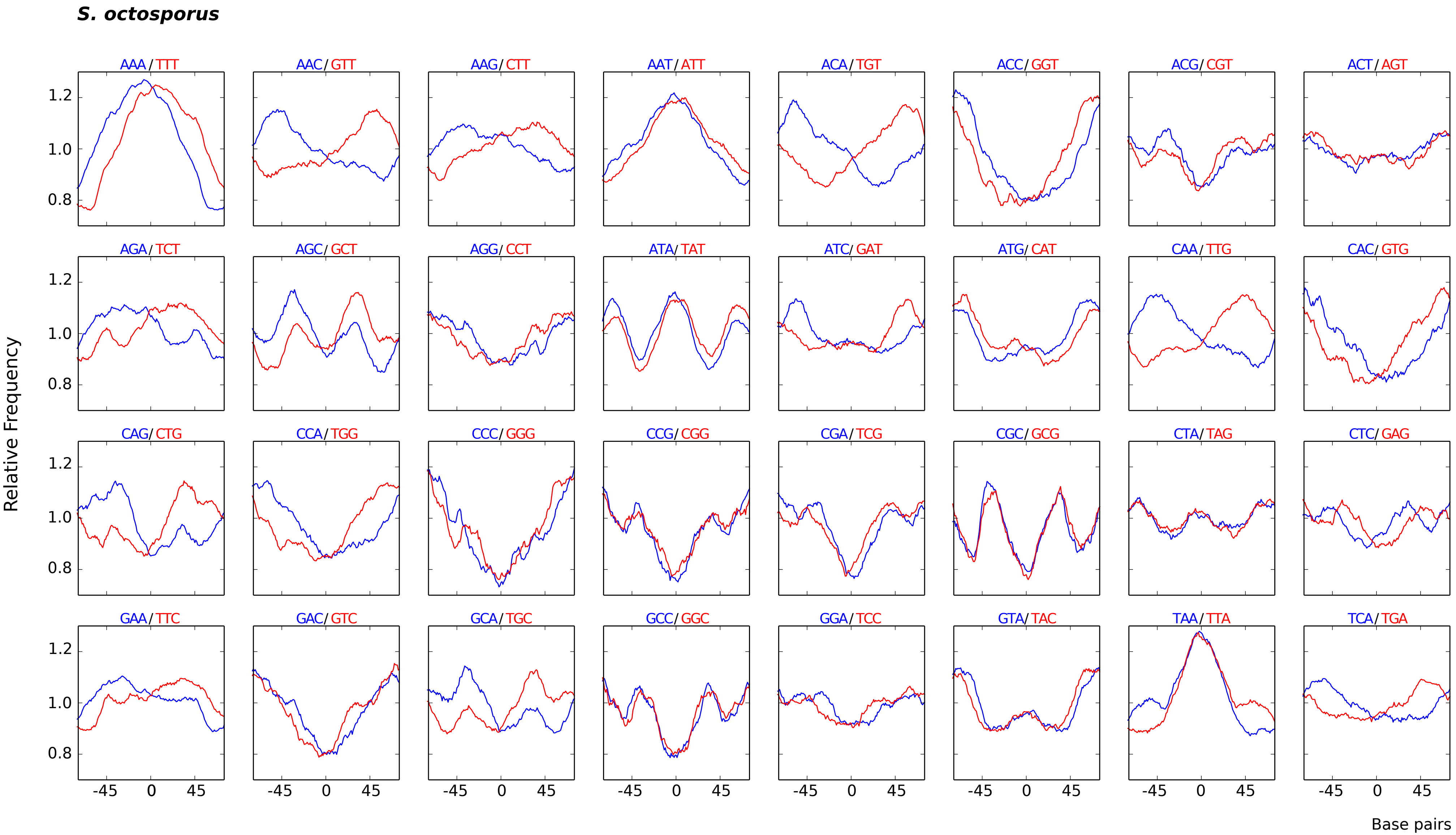

Supplementary Figure 5B

S. japonicus

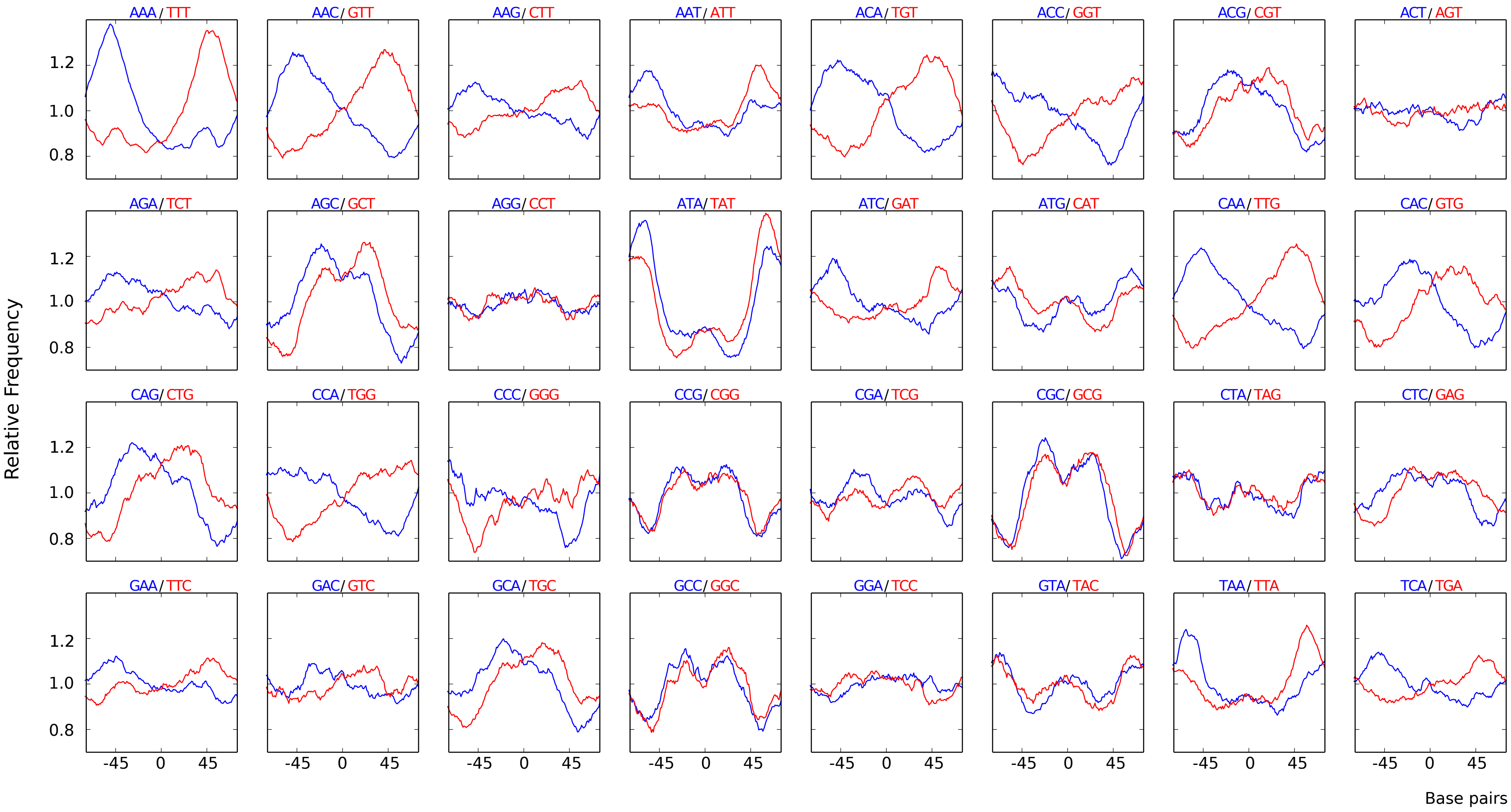

Supplementary Figure 5C

S. cerevisiae

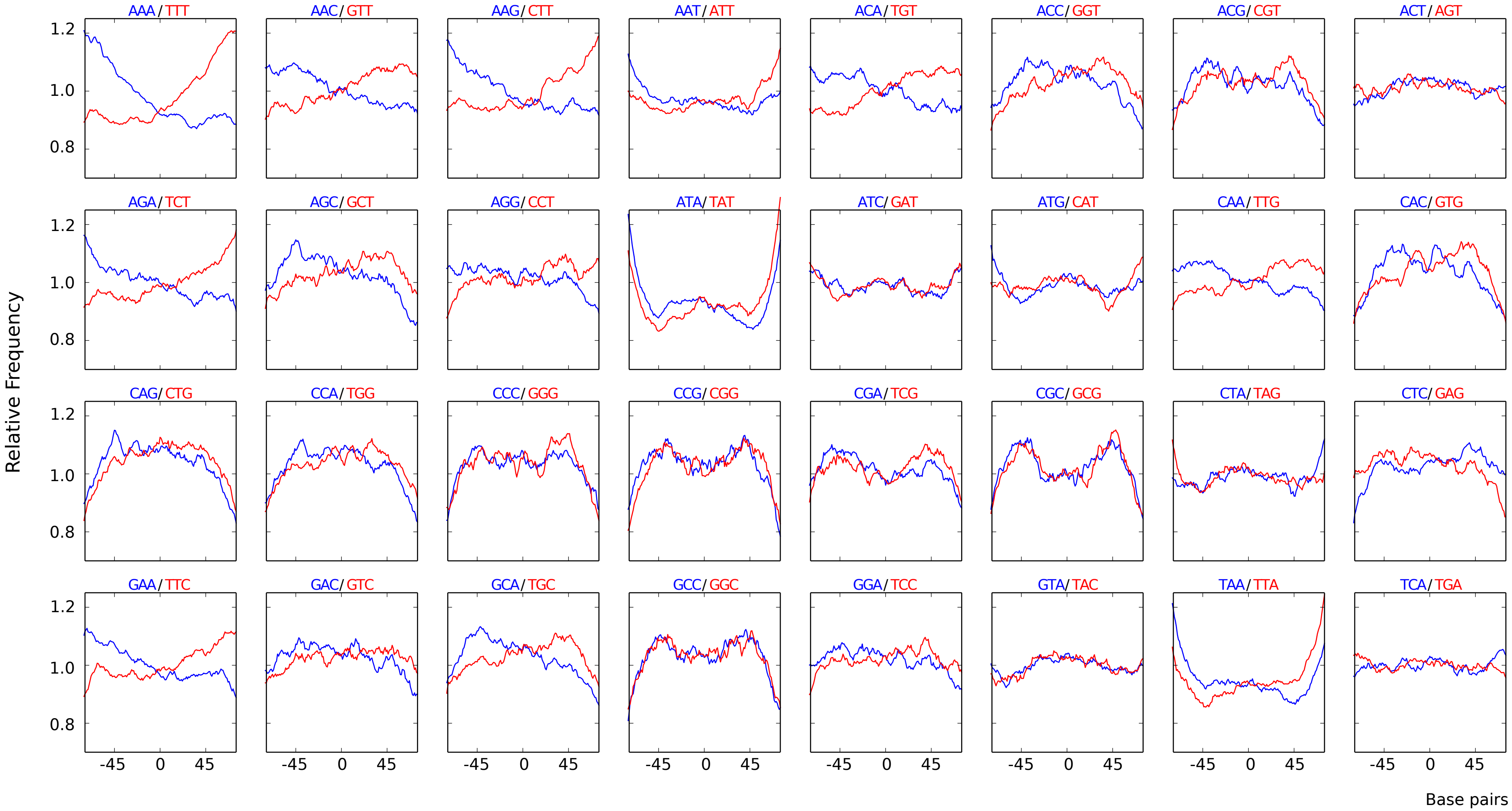

Supplementary Figure 5D

S. pombe

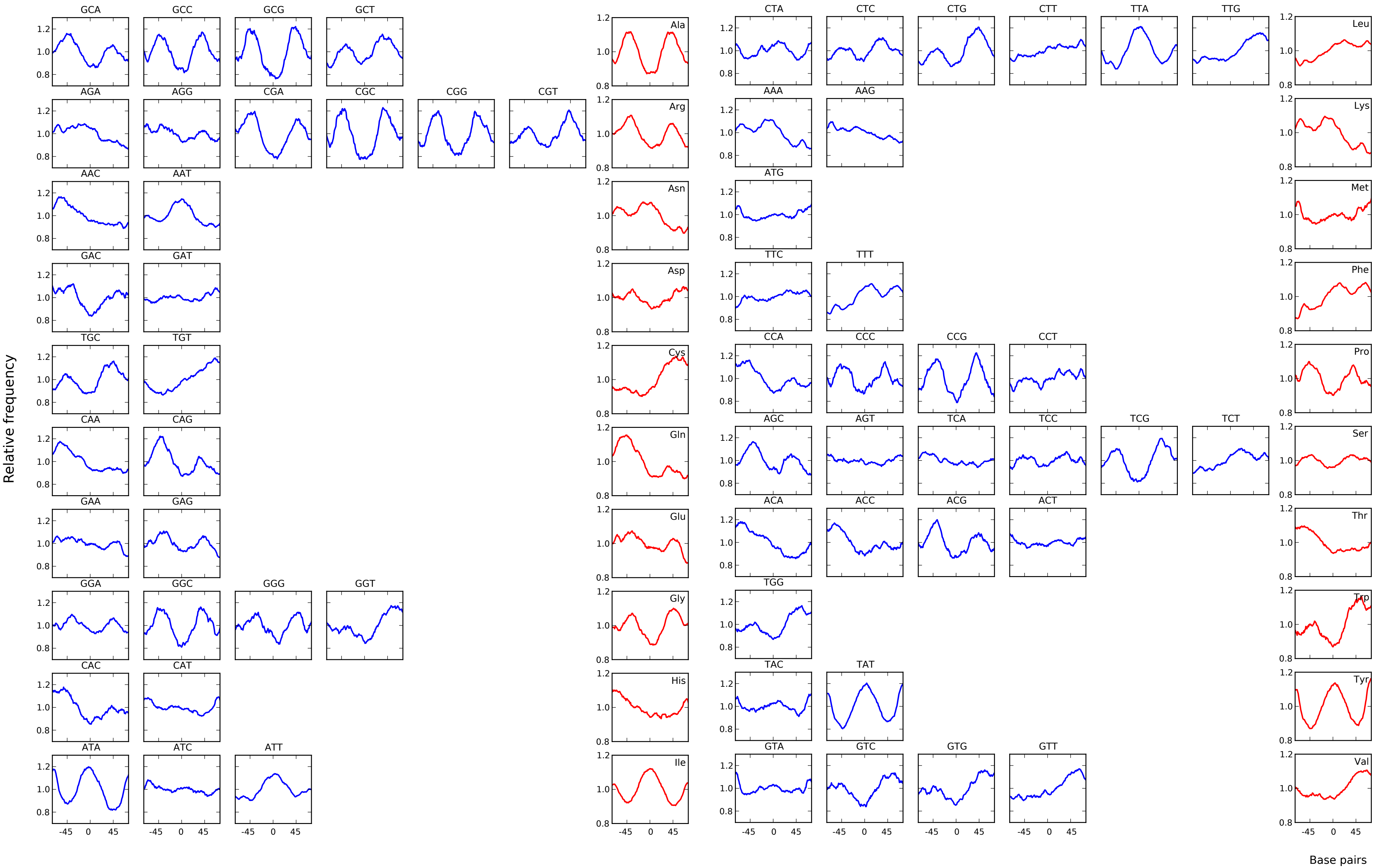

Supplementary Figure 6A

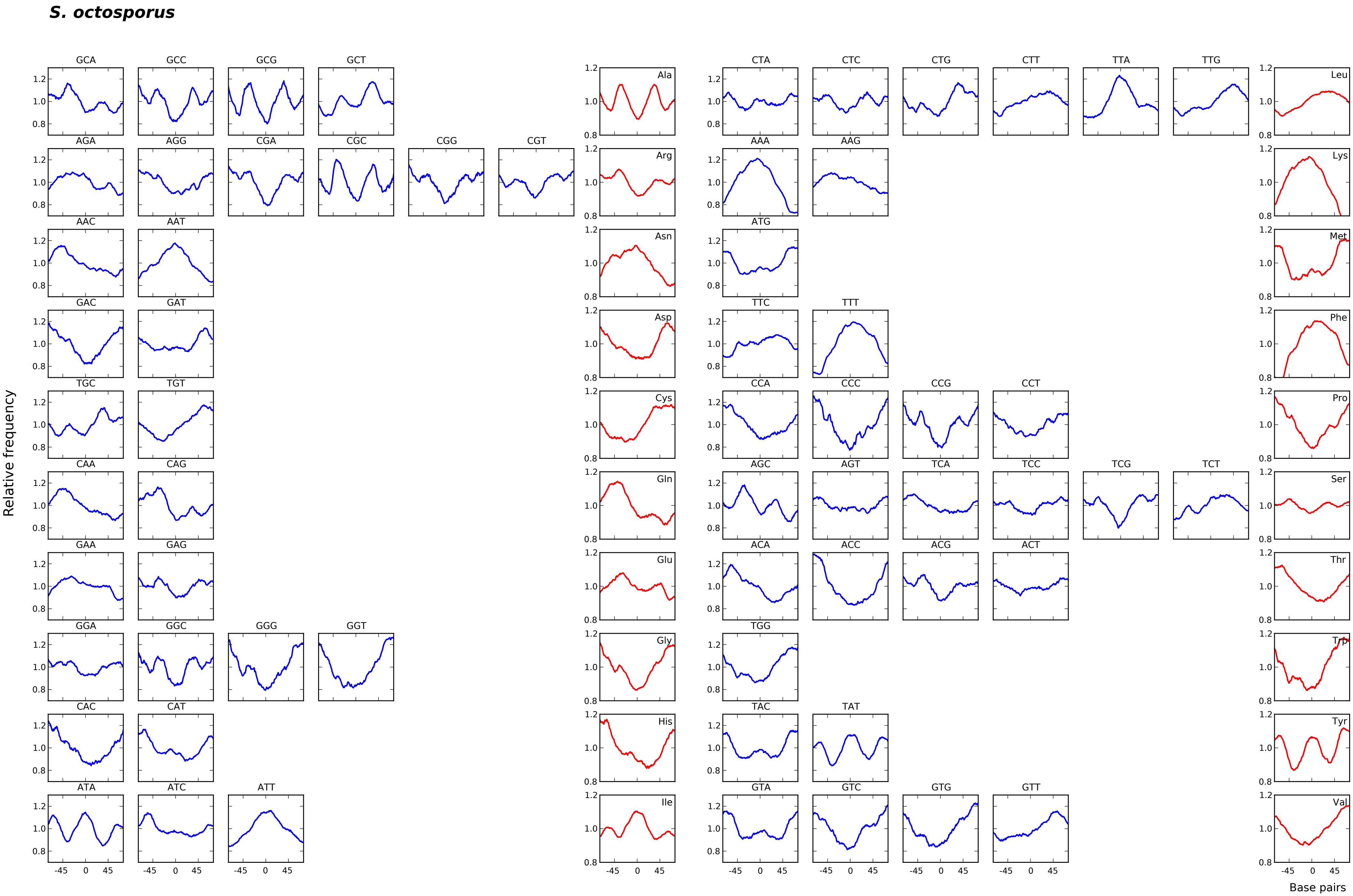

Supplementary Figure 6B

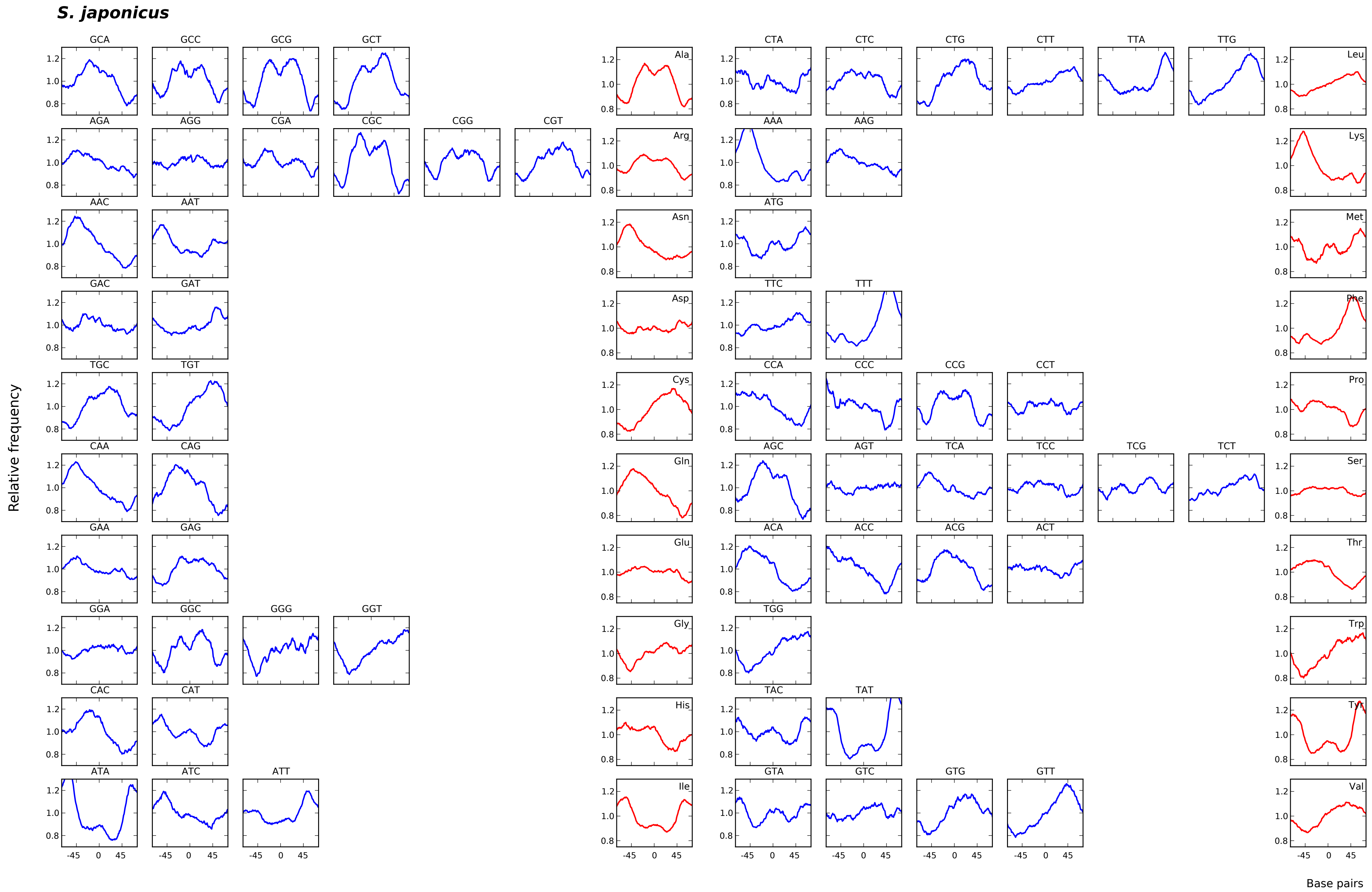

Supplementary Figure 6C

S. cerevisiae

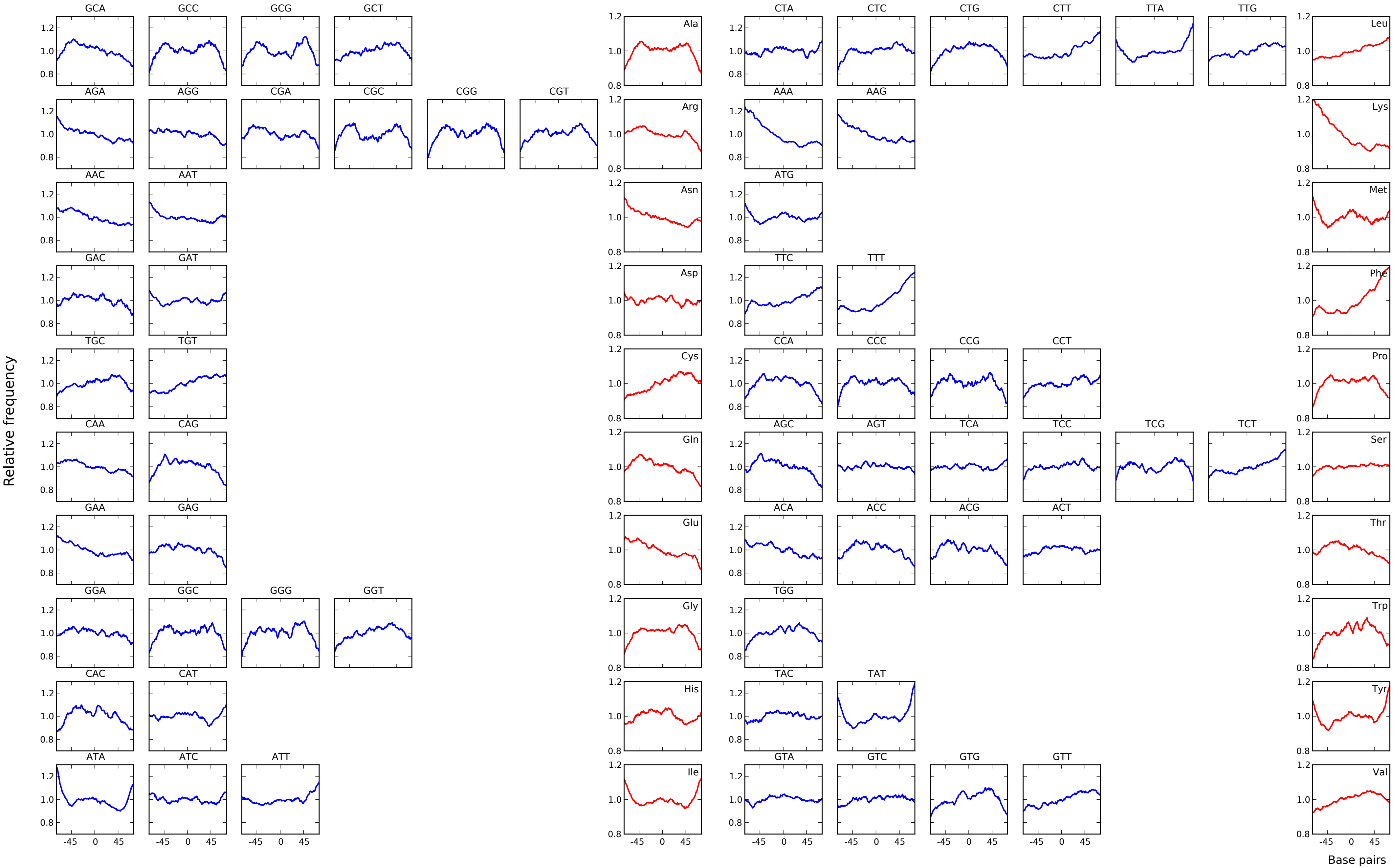

Supplementary Figure 6D

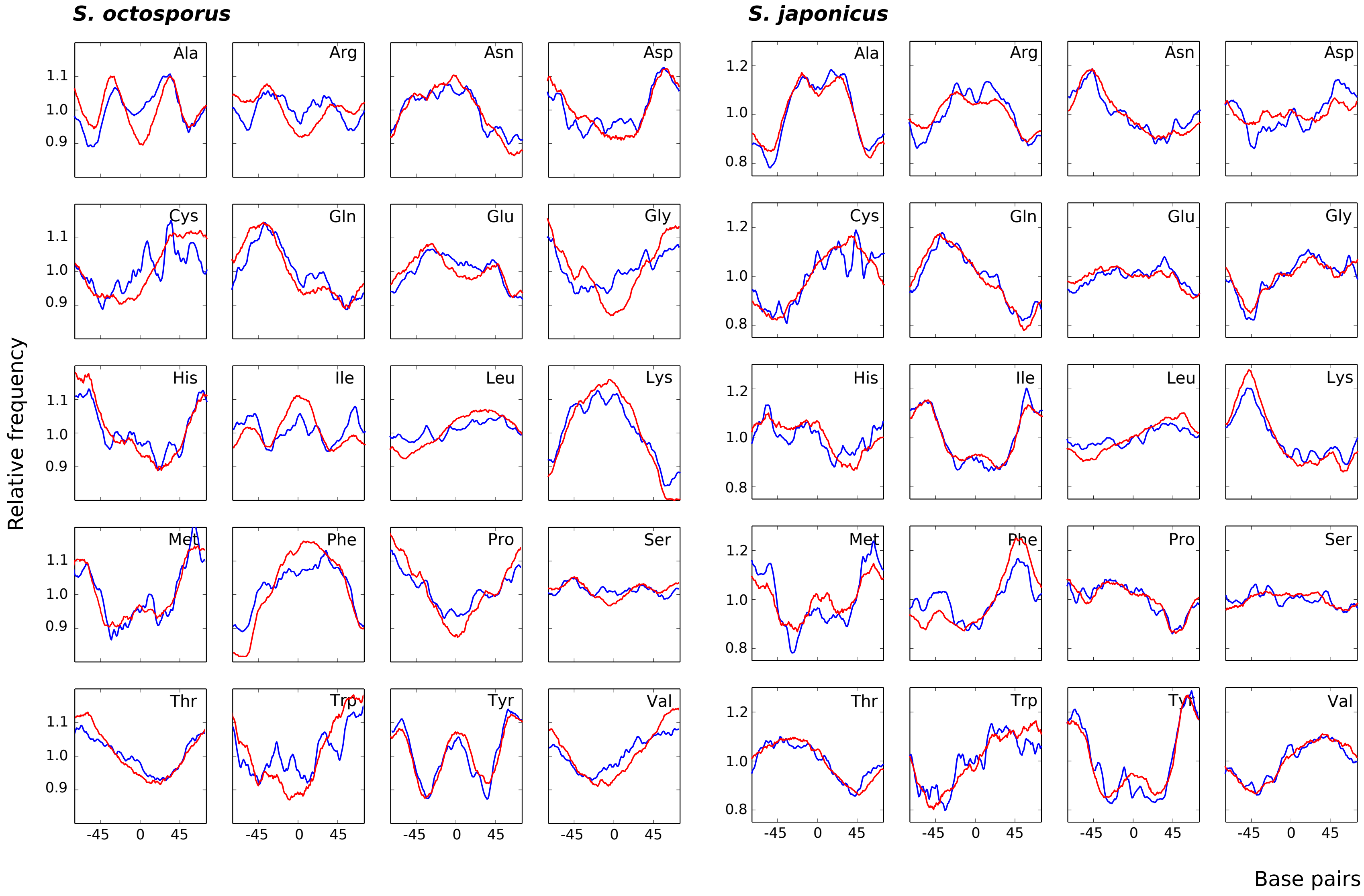

Supplementary Figure 7

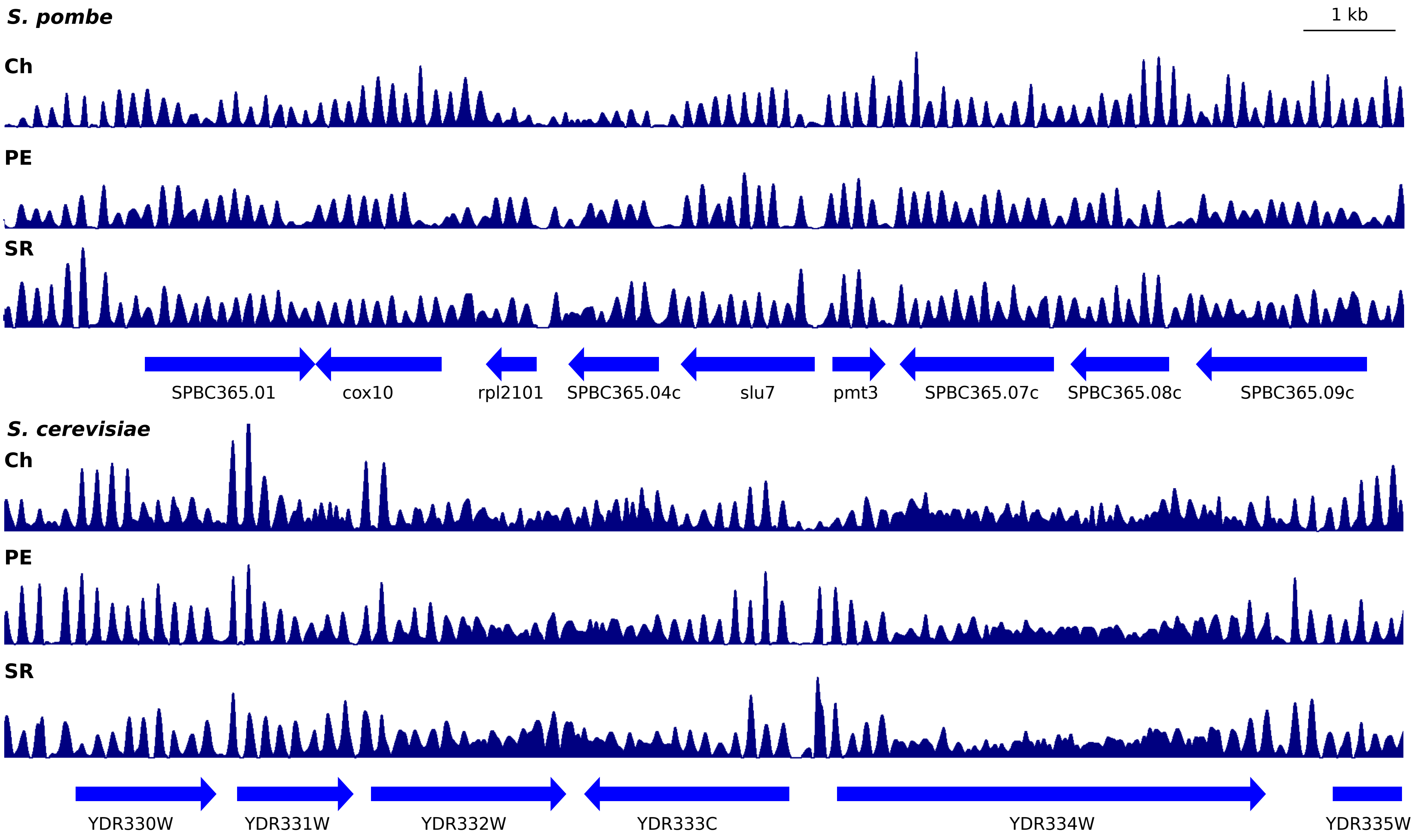

Supplementary Figure 8

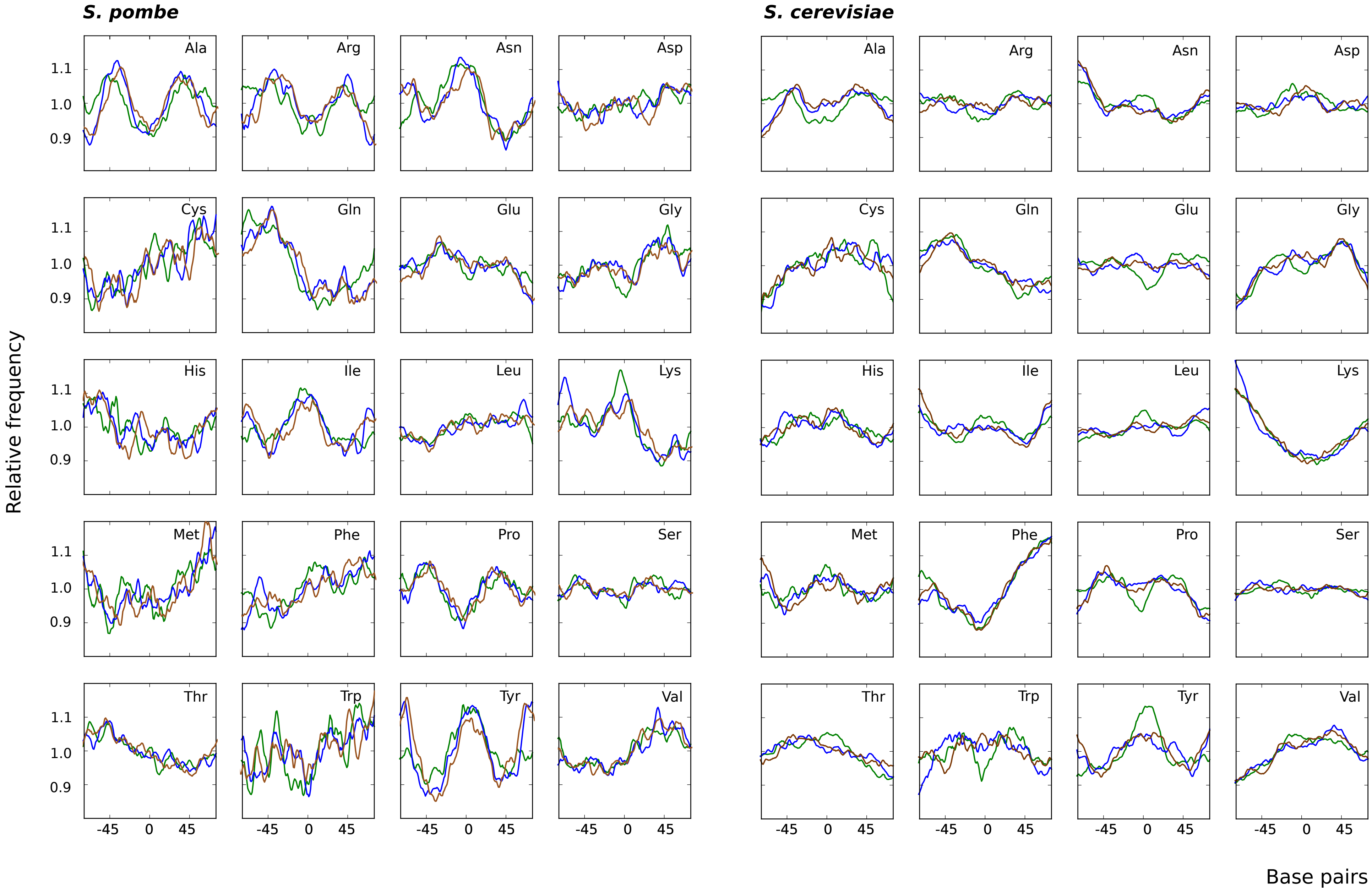

Supplementary Figure 9
